# Aktueller Stand der Durchführung von Nierenersatztherapien auf deutschen Intensivstationen

**DOI:** 10.1007/s00063-021-00835-y

**Published:** 2021-06-30

**Authors:** Carsten Willam, Melanie Meersch, Larissa Herbst, Peter Heering, Michael Schmitz, Michael Oppert, Stefan John, Achim Jörres, Alexander Zarbock, Uwe Janssens, Detlef Kindgen-Milles

**Affiliations:** 1 Medizinische Klinik 4, Universität Erlangen-Nürnberg, Ulmenweg 18, 91054 Erlangen, Deutschland; 2 Klinik für Anästhesiologie, operative Intensivmedizin und Schmerztherapie, Universitätsklinikum Münster, Münster, Deutschland; 3 Klinik für Nephrologie und Allgemeine Innere Medizin, Städtisches Klinikum Solingen, Solingen, Deutschland; 4 Zentrum für Notfall- und Intensivmedizin, Klinikum Ernst von Bergmann, Potsdam, Deutschland; 5 Klinikum Nürnberg, Medizinische Klinik 8 – Kardiologie, Paracelsus Medizinische Privatuniversität Nürnberg, Nürnberg, Deutschland; 6 Medizinische Klinik I, Köln-Merheim, Klinik für Nephrologie, Transplantationsmedizin und internistische Intensivmedizin, Köln, Deutschland; 7 Klinik für Innere Medizin und Internistische Intensivmedizin, St.-Antonius-Hospital, Eschweiler, Deutschland; 8 Klinik für Anästhesiologie, Universitätsklinikum Düsseldorf, Düsseldorf, Deutschland

**Keywords:** Akutes Nierenversagen, Nierenersatztherapie, Hämofiltration, Hämodialyse, Intensivmedizin, Acute kidney injury, Renal replacement therapy, Hemofiltration, Hemodialysis, Intensive care medicine

## Abstract

Eine akute Nierenschädigung (AKI) tritt heute bei 50 % aller kritisch kranken Patienten auf und etwa 15 % müssen mit einer Nierenersatztherapie (NET) behandelt werden. Obwohl eine NET ein häufiges und essenzielles Organersatzverfahren in der deutschen Intensivmedizin darstellt, ist es ist nicht bekannt, in welchem Umfang Nierenersatzverfahren zur Verfügung stehen, wer mit welcher Qualifikation eine NET durchführt, welche Formen der Antikoagulation verwendet werden und wie die Dosis der NET verschrieben wird. Die Deutsche Interdisziplinäre Vereinigung für Intensiv- und Notfallmedizin (DIVI) hat deshalb Ende 2019 unter ihren Mitgliedern eine Umfrage zu den strukturellen Gegebenheiten der NET in ihrem Arbeitsumfeld durchgeführt. Es konnten 897 Datensätze erfasst werden (31,1 % der Befragten), anhand derer die aktuellen strukturellen und prozeduralen Gegebenheiten bei der Durchführung der NET auf deutschen Intensivstationen beschrieben werden können. Es waren Krankenhäuser aller Versorgungsstufen vertreten, allerdings waren Krankenhäuser mit einer Bettenzahl von > 400 Betten (69,1 %) und Krankenhäuser der Schwerpunkt- und Maximalversorgung und Unikliniken (74,5 %) verstärkt vertreten. Kontinuierliche Nierenersatzverfahren stehen auf 93,3 % und intermittierende Verfahren auf 75,8 % der Intensivstationen in Deutschland zur Verfügung. Die Indikation zur NET wird in 91,9 % durch eine/n Facharzt/Fachärztin oder einen Facharzt/Fachärztin mit Zusatzweiterbildung Intensivmedizin und/oder Nephrologie gestellt. In Fragen der Therapiedurchführung sind jedoch Aspekte der Dialysedosis besser zu implementieren und dokumentieren.

## Abstract (en)

About 50% of all critically ill patients develop acute kidney injury (AKI) and approximately 15% receive renal replacement therapy (RRT). Although RRT is frequently used in intensive care units in Germany, it is currently unknown which RRT procedures are available, which qualification the involved staff has, which anticoagulation strategies are used and how RRT doses are prescribed. To investigate quality and structural characteristics of the performance of RRT in intensive care units throughout Germany, the German Interdisciplinary Society of Intensivists (Deutsche Interdisziplinäre Vereinigung für Intensiv- und Notfallmedizin [DIVI]) performed an inquiry among their members. A total of 897 members participated in the survey in which practical aspects were queried. In 69.1% of the cases, RRT was performed in hospitals with more than 400 beds and in 74.5% in university hospitals or other primary care hospitals. Furthermore, 93.3% of clinics are equipped with continuous and 75.8% with intermittent renal replacement devices. In 91.9%, indication for initiation of RRT was performed by trained physicians specialized in intensive care medicine or nephrologists. Intermittent as well as continuous modalities are both present in three-quarters of cases, which allows for individualized therapy. However, the documentation of dialysis dose needs to be improved.

## Hintergrund und Fragestellung

Die *akute Nierenschädigung* („acute kidney injury“ [AKI]) ist eine häufige Komplikation bei kritisch kranken Patienten mit erheblichen Auswirkungen auf die Morbidität und das Kurz- und Langzeitüberleben [7]. Die Inzidenz der AKI bei stationären Patienten liegt bei ca. 2 % in Krankenhäusern der Regelversorgung und steigt auf ca. 20 % in Kliniken der Maximalversorgung und Universitätskliniken [5]. Auf Intensivstationen kann die Inzidenz sogar mehr als 50 % betragen [3, 10]. Daher ist die Behandlung dieses Syndroms fester Bestandteil der Versorgung kritisch kranker Patienten. Die Ätiologie der AKI auf Intensivstationen ist multifaktoriell. Die Hauptursache ist die Sepsis (40 %), gefolgt von Hypovolämie, dem kardiogenen Schock und der Exposition mit Nephrotoxinen [3].

Die Nierenersatztherapie (NET) ist als rein supportive Maßnahme die einzige therapeutische Option bei der Behandlung der schweren AKI. Die Ziele der NET sind primär – und unabhängig von der zugrunde liegenden Ätiologie – die Kontrolle des Flüssigkeitsstatus, d. h. die Vermeidung oder die Behandlung einer Hypervolämie, die maschinelle Entgiftung zur Vermeidung der Urämie, der Ausgleich einer renalen Acidose und die Elektrolytkontrolle. Auf der anderen Seite stellt eine NET immer einen schweren Eingriff in die Homöostase des Organismus dar, die zu einem „dialytrauma“ führen kann. Die genaue Sachkenntnis des Verfahrens soll dieses „Dialytrauma“, das Aspekte wie Alkalose, Hypothermie, Hypophosphatämie, Imbalancen des Natrium-Chlorid-Haushalts, der Osmolalität und der Flüssigkeitsbilanzschwankungen umfasst, verringern.

Für die NET auf Intensivstationen stehen heute neben den kontinuierlichen Nierenersatzverfahren auch intermittierende prolongierte Verfahren („prolonged intermittent renal replacement therapy“ [PIRRT]) zur Verfügung. Darüber hinaus werden unterschiedliche Antikoagulationsstrategien eingesetzt, wie die regionale Zitratantikoagulation oder die systemische Antikoagulation mit unterschiedlichen Antikoagulanzien wie Heparin oder direkte Thrombininhibitoren wie Argatroban. Darüber hinaus sind neue Adsorbtions- und Aphereseverfahren verfügbar, die allein oder in Kombination mit einer NET eingesetzt werden können.

Diese Entwicklung stellt hohe Anforderungen an das medizinische Personal, das mit der Durchführung dieser Verfahren beauftragt ist. Aufgrund der Komplexität der intensivmedizinisch zu behandelnden Krankheitsbilder mit einem regelhaft auftretenden Mehrorganversagen kann die Anwendung solcher Verfahren heute nicht mehr isoliert unter dem Aspekt einer einfachen maschinellen Entgiftung betrachtet werden. Vielmehr muss zwingend ein integratives Therapiekonzept etabliert werden, in dem die NET als ein Bestandteil des „multi-organ support“ betrachtet wird und integraler Bestandteil der intensivmedizinischen Komplexbehandlung ist. So konnte eine aktuelle Untersuchung zeigen, dass in Deutschland mehr als 30 % der invasiv beatmeten Patienten und Patientinnen ein Nierenersatzverfahren benötigt [15]. Eine hohe Aktualität hat, dass bei schweren Verläufen einer COVID-19-Erkrankung ein AKI mit NET häufig auftritt. Das Management eines höhergradigen AKI macht es erforderlich, eine entsprechende Expertise zur NET vorzuhalten. Aus diesen Gründen sind eine profunde Sachkenntnis und Qualifikation der Therapierenden notwendig.

Mit dieser Umfrage der Sektion Niere der DIVI soll der aktuelle Stand der praktischen Durchführung der NET auf deutschen Intensivstationen erfasst werden. Auf dieser Grundlage können Empfehlungen zur Optimierung der Behandlung entwickelt werden.

## Studiendesign und Untersuchungsmethoden

Über die Plattform der DIVI wurde im Dezember 2019/Januar 2020 ein standardisierter Fragebogen an die Mitglieder versendet. Entwickelt wurde der Fragebogen durch die Sektion Niere der DIVI in Zusammenarbeit mit der DGIIN. Der Fragebogen enthielt 20 Fragen zu den strukturellen und prozeduralen Gegebenheiten auf den Intensivstationen der Teilnehmer (s. Anlage).

Die DIVI hatte zum Zeitpunkt der Befragung 2886 Mitglieder. Davon kamen 1173 (40,6 %) aus der Anästhesie, 440 (15,2 %) aus der inneren Medizin und 113 (3,9 %) aus der Neurologie/Neurochirurgie. Von den 2886 Mitgliedern kamen 905 (31,4 %) aus der Pflege bzw. waren nichtärztliche Mitarbeiter.

Die Auswertung erfolgte mit SPSS (Version 25, IBM Statistics, Deutschland).

## Ergebnisse

### Rücklaufquote und Demographie der Teilnehmer

Es konnten insgesamt 897 Datensätze ausgewertet werden, wodurch 31,1 % aller DIVI-Mitglieder erfasst wurden. In 50 Fällen (5,5 %) wurde angegeben, dass in der betreffenden Klinik kein Nierenersatzverfahren zur Verfügung steht und die Patienten mit Bedarf für eine NET verlegt werden. 74,5 % der Befragten waren Ärztinnen/Ärzte und 23,7 % Pflegekräfte. 76,4 % waren männlich, 23,6 % weiblich, wobei 83,7 % der Befragten mehr als 10 Jahre Intensiverfahrung aufwiesen. Nur 2,2 % gaben an, weniger als 5 Jahre Erfahrung zu haben. 63,2 % der befragten Ärztinnen/Ärzte hatten die Zusatzbezeichnung Intensivmedizin. 25,4 % der Pflegekräfte verfügten über die Fachweiterbildung Anästhesie und Intensivpflege.

### Kliniken und Intensivstationen

39,7 % der Befragten arbeiteten in Kliniken mit 200–600 Betten, 16,2 % mit 600–1000 Betten und 33,7 % mit über 1000 Betten. Es handelte sich bei 32,3 % um Universitätskliniken und bei 17,2 % um Krankenhäuser der Maximalversorgung. Nur 7,9 % der Befragten arbeiteten in einem Haus der Grundversorgung (Tab. 1 und 2).

**Table 1:** 

*Anzahl Betten*	*n*	*%*
Bis 200	94	10,4
201–400	184	20,5
401–600	172	19,2
601–1000	145	16,2
Über 1000	302	33,7
*Versorgungsstufen*	*n*	*%*
Grundversorgung	71	7,9
Regelversorgung	158	17,6
Schwerpunktversorgung	224	25
Maximalversorgung	154	17,2
Universitätskliniken	290	32,3
*Intensivstation Bettenzahl*	*n*	*%*
< 10	216	24
11–15	266	29,7
16–20	154	17,2
21–25	82	9,1
> 25	179	20
Kumulativ	*897*	*100*
*Verfahren verfügbar*	Verfahren angeboten	
Kontinuierliche Verfahren	790	93,3
Intermittierende Verfahren	642	75,8
SLEDD	303	35,8
Zytokinadsorber	360	42,5
Plasmapherese	491	58
Gesamt	*–*	*–*

**Table 2:** 

Maschinenanzahl (%)	0	1	2	3–5	> 5
Kontinuierliche Maschinen	9,1	11,9	23,5	29,5	26
Intermittierende Maschinen	19,8	14,4	23,5	23,7	18,5
SLEDD	64,6	5,3	6,4	11,5	12,3

Am häufigsten arbeiteten die Befragten (29,7 %) auf Intensivstationen mit 11–15 Betten. 46,3 % der Befragten gaben an, auf großen Intensivstationen mit über 15 Intensivbetten tätig zu sein (Tab. 1). Korrespondierend können die Intensivstationen der Befragten zu 57,2 % die erweiterte intensivmedizinische Komplexbehandlung (SuperSAPS) beanspruchen, bei 32,7 % wurde der Basissatz und in 10,1 % kein intensivmedizinischer Score abgerechnet.

**Figure 1:**
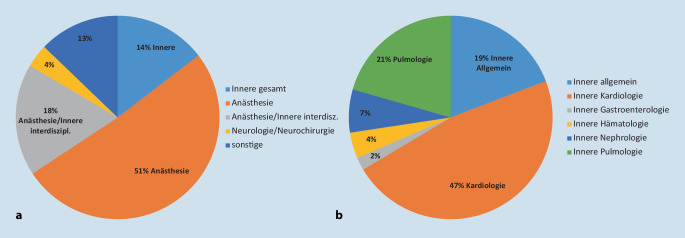

Knapp die Hälfte der Befragten gaben an, dass die Intensivstation unter der Leitung der Anästhesie geführt wird (51,1 %). 14,6 % der Intensivstationen wurden durch die innere Medizin, 17,8 % interdisziplinär (Anästhesie und innere Medizin), 3,7 % durch die Neurologie (12,8 % weitere Fachdisziplinen) geleitet (Abb. 1).

Der überwiegende Anteil (84,7 %) der Befragten gab an, dass die tägliche ärztliche Supervision durch einen Fach- oder Oberarzt mit der Zusatzbezeichnung Intensivmedizin erfolgt. Bei 14,6 % der Befragten erfolgte die ärztliche tägliche Visite durch einen Fach- oder Oberarzt ohne Zusatzbezeichnung Intensivmedizin.

### Diagnostik der AKI

Im Rahmen der Diagnostik der AKI gaben 96,0 % der Befragten an, dass 24/7 eine Labordiagnostik vorhanden sei. In 80,4 % der Befragten war eine Sonographie und in 93,4 % eine BGA Bestimmung auf der Intensivstation möglich. Bereits bei 7,9 % der Befragten waren neue Biomarkertests für die Bestimmung einer AKI verfügbar und bei 3,6 % ein elektronisches Alert-System. Nur 5,0 % der Befragten führten eine Urinmikroskopie auf der Intensivstation durch.

### Wer führt die Nierenersatztherapie auf der Intensivstation durch?

Bei 82,6 % der Befragten wurde die NET primär durch das eigene Intensivpersonal durchgeführt. Bei 373 der Befragten (44,0 %) konnte die NET ebenfalls durch eine hauseigene Dialyseabteilung bzw. einen Arzt aus der Nephrologie durchgeführt werden. Ein externer Dialyseanbieter wurde bei 12,8 % der Befragten hinzugezogen.

Kontinuierliche Nierenersatzverfahren wurden von 93,3 % der Befragten verwendet. Die intermittierenden Nierenersatzverfahren wurden von 75,8 % und verlängerte intermittierende Verfahren von 35,8 % der Befragten vorgehalten. Der Overlap lässt schließen, das größere Kliniken sowohl kontinuierliche (93,3 %) als auch intermittierende Verfahren (75,8 % der Befragten) im Sinne von individualisierten Strategien anbieten. Bei dem überwiegenden Anteil der Befragten (96,6 %) waren Nierenersatzverfahren rund um die Uhr (24/7) möglich. Die meisten Stationen hielten 3–5 Maschinen pro Station vor (Tab. 1).

Die Dokumentation der Dialyse in der Patientenkurve erfolgte bei 50,6 % der Befragten elektronisch. Ein explizites Dialyseprotokoll verwendeten 72,5 % in Papierform und 21,6 % in elektronischer Form.

Hinsichtlich der Frage, wer auf der Intensivstation ein Nierenersatzverfahren indiziert, gaben 67,8 % der Befragten an, dass Intensivmediziner mit der Zusatzweiterbildung Intensivmedizin und in 18,1 % Nephrologinnen/Nephrologen die Indikation stellten. Bei 46,6 % der Befragten wurde die Indikation interdisziplinär gestellt (Tab. 3).

**Table 3:** 

*Indikationsstellung während der Dienstzeiten*	%	
Assistenzarzt Intensivstation	24,8	
Facharzt ZWB Intensivmedizin	67,8	
Assistenzarzt Nephrologie	3,1	
Facharzt Nephrologie	18,1	
Externer Nephrologe	6	
Intensivmediziner/Nephrologen interdisziplinär	46,6	
*Indikationsstellung durch Nichtnephrologen (41,1* *%)*	%	
Anästhesie	70,5	
Neurologie/Neurochirurgie	3,6	
Unfallchirurgie	1,9	
Herzchirurgie	4,2	
Abdominalchirurgie	3,1	
Sonstige	16,7	
*Aufbau und Betrieb des Nierenersatzverfahren***s**	Aufbau, %	Betrieb, %
Assistenzarzt der Intensivstation	20	34,5
Facharzt	9,7	21
Pflegepersonal der Intensivstation	32,8	55,3
Dialysepersonal intern	30,3	15
Dialysepersonal extern	8	8,5

Die Aufrüstung der Maschinen wurde zu ungefähr je einem Drittel durch das Pflegepersonal der Intensivstationen (32,8 %), durch Ärztinnen/Ärzte der Station (29,7 %) oder durch internes Dialysefachpersonal (30,3 % der Befragten) durchgeführt (Tab. 3).

### Durchführung der Nierenersatztherapie

**Figure 2:**
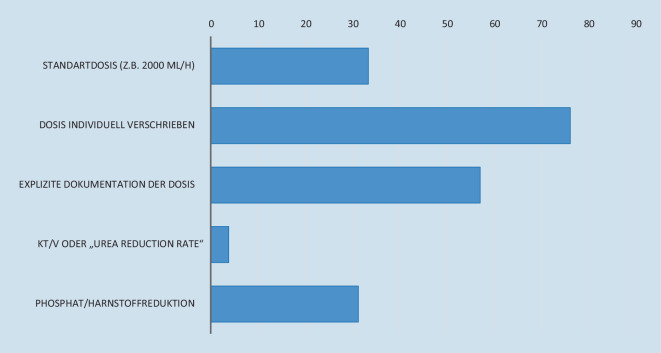

In der Umfrage gaben 76,2 % der Befragten an, dass eine individuelle Dosis verschrieben und explizit dokumentiert wird. 33,4 % der Befragten verwendeten eine Standarddosis von z. B. 2000 ml/h bei kontinuierlichen Verfahren (Abb. 2).

**Figure 3:**
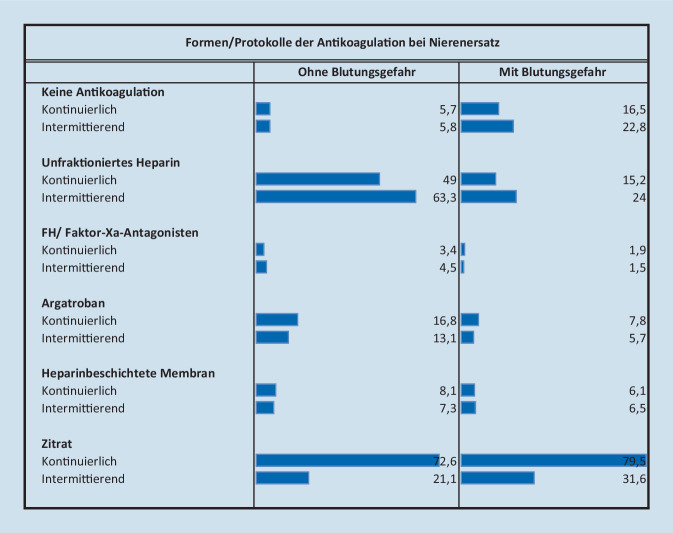

Als Antikoagulation hielten die meisten Befragten unfraktioniertes Heparin (81,3 %) und eine regionale Zitratantikoagulation (RCA; 93,6 %) vor. Bei den intermittierenden Verfahren wurde überwiegend unfraktioniertes Heparin verwendet (63,3 % der Befragten). Bei kontinuierlichen Verfahren wurde am meisten RCA verwendet (72,6 % der Befragten), auch wenn keine Blutungsgefahr vorliegt. Bei Blutungsgefahr verwendeten 79,5 % der Befragten die RCA für das kontinuierliche Nierenersatzverfahren und 16,5 % der Befragten gaben an, dann keine Antikoagulation durchzuführen (Abb. 3).

## Diskussion

Diese Umfrage wurde unter den Mitgliedern der DIVI – der größten intensivmedizinischen Fachvereinigung in Deutschland – durchgeführt. Mit einer Rücklaufquote von 33,1 % und 897 auswertbaren Fragebögen ist dies derzeit die aktuellste und umfangreichste Studie zur Erfassung der strukturellen und prozeduralen Gegebenheiten zur Behandlung der AKI und zur Durchführung der NET auf deutschen Intensivstationen.

Bei der Wertung der Ergebnisse muss berücksichtigt werden, dass unter den Antwortbögen Kliniken der Maximalversorgung und Universitätskliniken mit einem Anteil von fast 49,5 % stark vertreten sind. Dementsprechend hatten auch 29,1 % der Intensivstationen mehr als 20 Betten, was ebenfalls auf die hohe Teilnahmequote größerer Kliniken hindeutet (Tab. 1). Das Antwortverhalten kann jedoch unter Einschränkung auf der anderen Seite auch die Vermutung stützen, dass NET primär durch größere Häuser durchgeführt wird.

Die Leitung der Intensivstationen und damit die Zuordnung zu einer primären Fachdisziplin ergab, dass etwa die Hälfte aller Intensivstationen unter anästhesiologischer Leitung steht. Die internistisch geführten Intensivstationen werden etwa zu 47 % kardiologisch, 21 % pulmonologisch und zu etwa 7 % nephrologisch geführt.

Bei fast allen Befragten war eine Basisdiagnostik mit den relevanten Laborwerten sowie eine Blutgasanalytik 24/7 verfügbar. Hier zeigt sich durchwegs eine gute Versorgungsqualität. Die Sonographie, unter anderem essenziell zum raschen Ausschluss einer postrenalen AKI, war hingegen nur bei 80,4 % der Befragten direkt auf der Intensivstation vorhanden.

Eine Urinmikroskopie wird aktuell in den meisten Kliniken in den Zentrallaboren und nicht „on-site“ durchgeführt. Sie kann der raschen Diagnostik einer akuten Tubulusnekrose, einer Harnwegsinfektion sowie als Diagnostikum von Intoxikationen (z. B. Oxalatkristalle bei Ethylenglykolvergiftung) oder einer Vaskulitis oder Glomerulonephritis dienen [2, 13]. Die On-site-Urinmikrokopie ist in der Akutdiagnostik der AKI auf Intensivstationen eher unterrepräsentiert.

Bemerkenswert ist, dass 7,9 % der Befragten bereits neue Biomarker (z. B. NGAL, TIMP2/IGFBP7) zur Diagnostik der AKI verwenden. Derzeit wird diskutiert, ob eine Neudefinition der KDIGO-Stadien der AKI unter Einbeziehung neuer Biomarker sinnvoll wäre [8]. In diesem Fall würden neue Biomarker deutlich größere Bedeutung bei der AKI-Diagnostik erhalten und müssten in die Routinediagnostik integriert werden.

Insgesamt zeigt sich eine gute Verfügbarkeit der diagnostischen Maßnahmen auf den befragten Intensivstationen. Eine Steigerung der Verfügbarkeit der Sonographie wäre wünschenswert. Der Beginn einer NET ohne den vorherigen Ausschluss eines postrenalen Abflusshindernisses bleibt inakzeptabel. Anzumerken ist, dass, wenn auch die On-site-Verfügbarkeit der Sonographie nicht vollständig ist, es dennoch plausibel erscheint, dass in jedem Akutkrankenhaus eine Sonographie dennoch zeitnah verfügbar ist.

Bei der Indikationsstellung für eine NET spiegeln sich mutmaßlich die Leitungsstrukturen der größeren Kliniken wieder, in der Intensivstationen durch erfahrene Intensivmediziner mit ZWB Intensivmedizin und mehr als 10 Jahren Berufserfahrung geleitet werden. 70,5 % der Anästhesistinnen/Anästhesisten stellen die primäre Indikation zur NET. In 46,6 % der Kliniken wird eine interdisziplinäre Entscheidung durch Intensivpersonal und die Nephrologie etabliert.

Der überwiegende Anteil der Stationen kann eine NET selbst durchführen (82,6 %) und hält diese auch rund um die Uhr vor. Zu 44,0 % sind in der Durchführung hauseigene sowie zu 12,8 % externe Nephrologinnen/Nephrologen beteiligt. Vielerorts ist traditionellerweise die Nephrologie bei der Durchführung der intermittierenden Verfahren involviert, wohingegen kontinuierliche Verfahren überwiegend durch das Personal der Intensivstation durchgeführt werden. Dieses Konzept ist nicht spezifisch für Deutschland, sondern ist z. B. auch in den USA zu finden [1]. Die Umfrage zeigt daher insgesamt eine erhebliche Interdisziplinarität beim Einsatz von NET auf den Intensivstationen.

Die Geräteausstattung der Intensivstationen ist nach unseren Ergebnissen gut. Auf über 90,9 % der erfassten Intensivstationen waren Geräte zur kontinuierlichen, auf 80,2 % Geräte zur intermittierenden Therapie und bei 35,4 %, also ca. einem Drittel, wird ebenfalls eine PIRRT als Behandlungsoption angeboten. Dementsprechend ist davon auszugehen, dass an den meisten Standorten eine individualisierte Therapie durchführbar ist. Erstaunlich ist, dass derzeit trotz der noch schwachen Evidenz etwa 42,5 % der Befragten eine Zytokinadsorption vorhalten.

Zur Antikoagulation des extrakorporalen Kreislaufs sind in den meisten Kliniken mehrere Verfahren verfügbar. In Deutschland wird im internationalen Vergleich überproportional häufig eine regionale Zitratantikoagulation (RCA) für Patienten mit sowie ohne Blutungsgefahr eingesetzt. Diese Entwicklung ist insbesondere im operativen Bereich verständlich, da die RCA das Blutungsrisiko und die Transfusionsrate reduziert und gleichzeitig zu einer verlängerten Filterstandzeit führt [11]. Andererseits zeigt es sich, dass nicht nur die Rate an metabolischen Komplikationen (schwere Hypokalzämien, schwere Hypophosphatämien, metabolische Alkalosen), sondern auch die der Infektionen unter RCA signifikant erhöht ist [14]. Für die intermittierende NET sind Heparine weiter der Standard (63,3 % der Befragten).

Die aktuellen Leitlinien der KDIGO und der nationalen Fachgesellschaften empfehlen für die kontinuierliche NET eine Dosis, definiert als „total effluent“ (Summe Dialysat plus Ultrafiltrat), von 20–25 ml/kg und Stunde. Um dieses Ziel zu erreichen, wird empfohlen eine Dosis von 25–30 ml/kg und Stunde zu verschreiben, da durch Unterbrechungen des Verfahrens durch Clotting, Transporte und andere Ursachen die tatsächlich applizierte Dosis immer geringer als die verschriebene Dosis ist. Das Dosisziel wird dann nicht erreicht und eine Unterdialyse mit nachfolgend erhöhter Letalität kann resultieren [4, 12].

Etwa 76,2 % der Befragten gaben an, dass die Dosis individuell verschrieben wird, allerdings antworteten 33,4 %, dass die Standarddosis 2000 ml/h sei. Hier besteht eine offensichtliche Diskrepanz. Bei einem angenommenen Körpergewicht zwischen 60 und 90 kg entsprächen 2000 ml/h tatsächlich in etwa der empfohlenen Dosis von 25 ml/kg und Stunde. Bei einem höheren Körpergewicht entstünde hier jedoch eine Unterdosierung. Eine explizite Dokumentation der applizierten Dosis erfolgt nur in 57,1 % der Fälle. Eine Überprüfung der Dialyseeffektivität erfolgt immerhin noch in 31,1 % mittels Phosphat- oder Harnstoffreduktion. Das Kt/V als eine Dosisangabe aus dem chronischen Dialysebereich ist im Akutbereich nicht etabliert [4]. Zur Optimierung der individualisierten Dosis einer NET sollten die verschriebene und die applizierte Dosis stets dokumentiert werden und Laborkontrollen – z. B. mittels der Harnstoffreduktionsrate – erfolgen [6]. Damit kann einerseits eine hinreichende Urämiekontrolle ermöglicht werden, andererseits jedoch ein „dialytrauma“ mit einer Überdialyse oder Filtration vermieden werden [9].

### Schwächen.

Von der Zahl der Antworten kann nur bedingt auf die Zahl der beteiligten Kliniken rückgeschlossen werden, da mehrere Mitarbeiter aus einer Klinik geantwortet haben könnten. Es liegt eine hohe Repräsentation der größeren Kliniken vor, wobei zu berücksichtigen ist, dass maschinelle Organersatzverfahren weiterhin eine Domäne der Maximalversorger und Universitätskliniken darstellen. Weiterhin entspricht die Verteilung der Antwortenden auf die wichtigsten intensivmedizinischen Fachdisziplinen der Mitgliederstruktur der DIVI und nicht zwangsläufig der Leitungsstrukturen der deutschen Intensivmedizin.

### Stärken.

Diese Studie ist die aktuellste und umfangreichste Untersuchung zur praktischen Durchführung der NET auf deutschen Intensivstationen. Es wurden 31,1 % aller DIVI Mitglieder erfasst, sodass ein guter Überblick über die derzeitige Situation in Deutschland vermittelt werden kann. Es werden alle relevanten Aspekte im Umfeld der AKI erfasst, sodass hierdurch Empfehlungen zur Optimierung für die Praxis entwickelt werden können.

## Fazit für die Praxis

- Diese Studie liefert einen guten Überblick über die Verfügbarkeit von Nierenersatzverfahren auf deutschen Intensivstationen. Die meisten Kliniken halten eine Nierenersatztherapie vor. Nur in 5 % der Fälle müssen Patienten dazu in ein anderes Krankenhaus verlegt werden.
- In den größeren Krankenhäusern ist eine kontinuierliche NET fast flächendeckend verfügbar, intermittierende Verfahren bei 76 %.
- Neue Biomarker und elektronische Alerts spielen in Deutschland aktuell eine noch untergeordnete Rolle. Eine Sonographiediagnostik in nur 80 % „on-site“ verfügbar.
- Eine individualisierte Nierenersatzdosis wird nur durch etwa die Hälfte der Befragten eingesetzt. Die regionale Zitratantikoagulation ist in 72,6 % der befragten Häuser für kontinuierliche Nierenersatzverfahren verfügbar und wird häufig als Standardverfahren verwendet.
- Die Indikation für eine Nierenersatztherapie wird überwiegend durch Intensivmediziner durchgeführt. Der Ausbildungsstand der behandelnden Ärztinnen/Ärzte ist gut. Die die Nierenersatztherapie indizierenden Intensivmedizinerinnen/Mediziner haben zu einem großen Teil die Zusatzweiterbildung Intensivmedizin.
